# Cochlear Homeostasis in Sensorineural Hearing Loss: Mechanisms, Implications, and Therapeutic Prospects

**DOI:** 10.3390/ijms27010102

**Published:** 2025-12-22

**Authors:** Srdjan M. Vlajkovic, Haruna Suzuki-Kerr, Bryony A. Nayagam

**Affiliations:** 1 Department of Physiology, Faculty of Medical and Health Sciences, The University of Auckland, Auckland 1023, New Zealand; h.suzuki-kerr@auckland.ac.nz; 2 Eisdell Moore Centre, Faculty of Medical and Health Sciences, The University of Auckland, Auckland 1023, New Zealand; 3 Department of Audiology and Speech Pathology, The University of Melbourne, Parkville, VIC 3010, Australia; b.nayagam@unimelb.edu.au

**Keywords:** cochlea, homeostasis, stria vascularis, sensorineural hearing loss, gene therapy, regenerative medicine

## Abstract

Cochlear homeostasis is critical for the preservation of hearing sensitivity by maintaining optimal cochlear fluid composition, sustaining electrochemical gradients, and supporting the function of sensory and supporting cells in the cochlea. Sensorineural hearing loss, resulting from the damage or loss of sensory hair cells, auditory neurons and other cochlear cells and structures, is intimately linked to disruptions in the homeostatic environment. In this narrative review, we explore the cellular and molecular pathways underpinning cochlear homeostasis in health and disease and examine the mechanisms by which failed homeostasis leads to sensorineural hearing loss. We further discuss current research avenues and emerging therapeutic strategies to restore or compensate for the loss of homeostatic balance. These interventions suggest a future where regenerative healing is possible, ultimately leading to permanent repair and functional recovery.

## 1. Introduction

**Figure 1:**
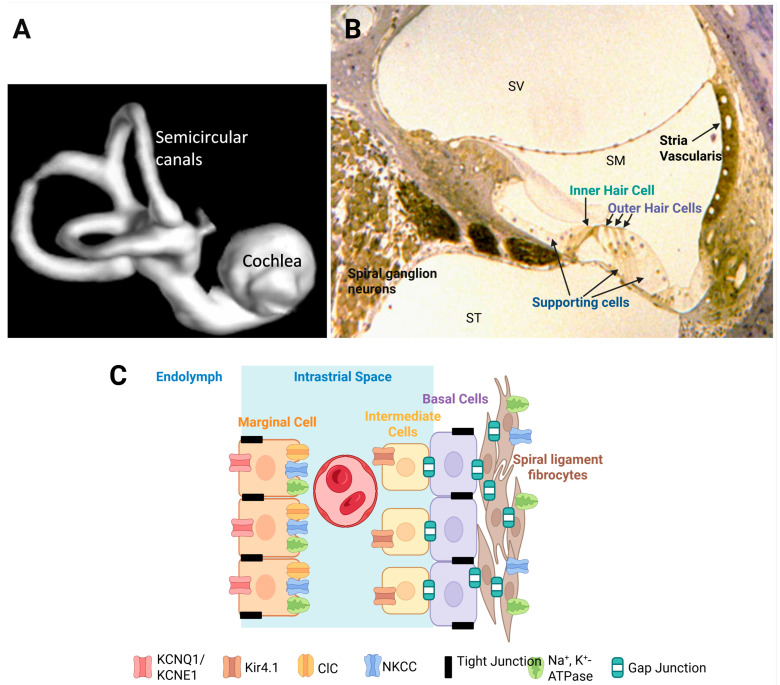
The structure of the mammalian inner ear. (**A**) MRI of the human inner ear. The inner ear contains two sensory organs, the hearing organ (cochlea) and the vestibular organ, which includes semicircular canals and two otolith organs (utricle and saccule). (**B**) The cochlea comprises three fluid-filled compartments: Scala vestibuli (SV) and scala tympani (ST) filled with perilymph, a typical extracellular fluid with high Na^+^ concentration, and scala media (SM) filled with K^+^-rich endolymph. The organ of Corti is the sensory organ of the cochlea. It contains two types of sensory cells, inner hair cells and outer hair cells, upheld by supporting cells, such as Deiters’, Hensen’s, pillar, inner border, inner phalangeal and inner sulcus cells. (**C**) Stria vascularis is the secretory epithelial tissue of the cochlea composed of marginal, intermediate and basal cells. The endocochlear potential (+80–100 mV), the driving force for sensory transduction, is generated by a network of gap junctions (e.g., connexin 26, connexin 30), K^+^ channels (KCNQ1/KCNE1, Kir 4.1), ion transporters (NKCC1, Na^+^/K^+^-ATPase) and chloride ion channels (CIC) in the stria vascularis. K^+^ recycling from endolymph back to the stria vascularis is also supported by ion transporters and gap junctions in the spiral ligament fibrocytes. Created in BioRender. Suzuki-Kerr, H. (2025) https://BioRender.com/se9jjmc (accessed on 2 November 2025).

The human ear is a biological and evolutionary masterpiece, responsible for converting sound vibrations into electrical signals that the brain interprets as sound. The central structure of the auditory system is the cochlea of the inner ear (Figure 1A), a spiral-shaped organ designed for exquisite sensitivity to sound intensity and frequency discrimination [1]. Maintaining the delicate balance of the cochlear environment, known as cochlear homeostasis, is critical for optimal auditory function. Yet, disruptions in this equilibrium are key to sensorineural hearing loss, affecting millions worldwide.

Sensorineural hearing loss (SNHL) encompasses a range of pathologies in which the cochlear sensory hair cells, supporting structures, and synaptic connections to the auditory nerve are compromised [2]. Unlike conductive hearing loss, which impedes sound transmission through the outer or middle ear, SNHL is caused by cellular and molecular alterations in the cochlea due to altered homeostasis.

The term “electrochemical homeostasis” refers to the maintenance of ion gradients in the cochlea, specifically potassium (K^+^) and sodium (Na^+^) levels, and the regulation of fluid composition, particularly the differential ionic makeup of the endolymph and perilymph [3]. Endolymph, which fills the scala media, is rich in K^+^ and low in Na^+^. In contrast, the perilymph contained in the scala tympani and scala vestibuli is a typical extracellular fluid, characterised by high Na^+^ and low K^+^ levels. This electrochemical separation facilitates rapid transduction of mechanical stimuli into receptor potentials and, consequently, the generation of nerve impulses in the auditory nerve.

Even subtle deviations in ionic composition or fluid balance can have a profound impact on hair cell function. Multiple factors, including genetic predisposition, ageing, noise exposure, ototoxic drug use, and metabolic disturbances, can perturb this fine balance. The dysregulation of cochlear homeostasis ultimately results in cell damage, apoptosis, and permanent SNHL [4,5].

This narrative review first delves into the cellular and molecular mechanisms that maintain homeostasis within the cochlea. We will then explore how disruptions in these mechanisms give rise to SNHL and discuss the complex interplay between environmental insults and the cochlear response to stress and injury. Finally, we will consider promising therapeutic interventions, ranging from pharmacological and gene therapies to regenerative strategies, to restore the cochlear environment and restore function. By comparing cochlear homeostasis in health and disease, we aim to review the latest research on the molecular mechanisms and treatments for SNHL, from basic science to clinical innovation.

## 2. Structural Organisation of the Cochlea

The bony labyrinth of the cochlea contains three fluid-filled compartments: the scala vestibuli, scala media, and scala tympani (Figure 1B). The scala media houses endolymph, a K^+^-rich fluid that fills the space around the apical surfaces of the sensory hair cells and supporting cells in the organ of Corti [1]. In contrast, the scala vestibuli and scala tympani contain perilymph, which has an ionic composition similar to cerebrospinal fluid with high Na^+^ and low K^+^ levels. The distinct ionic compositions between these fluids underlie the endocochlear potential (EP), a key driving force for sensory transduction [6].

The organ of Corti contains organised rows of inner and outer hair cells, each hair cell type serving specific roles in sound detection and amplification (Figure 1B). Inner hair cells are the primary sensory cells sending sound information to the brain, whereas outer hair cells act as biological amplifiers by mechanically enhancing sound vibrations of the basilar membrane through electromotility [7,8].

The highly organised structure of the organ of Corti is further upheld by a network of supporting cells, including Deiters’ cells, pillar cells, and Hensen’s cells [1]. These cells provide both mechanical reinforcement and metabolic support to the sensory hair cells. They also contribute to K^+^ recycling, thus maintaining the consistent ionic concentration in endolymph necessary for sensory transduction [9]. Tight junctions between the sensory and supporting cells of the organ of Corti [10] form a high-resistance paracellular barrier (the reticular lamina) that prevents uncontrolled ion and fluid movement between endolymph and perilymph. Due to the apical position of tight junctions, the apical surfaces of sensory hair cells are bathed in endolymph while the basolateral sides are bathed in perilymph. This barrier is crucial for maintaining the ionic gradients that underlie the endocochlear potential and mechanoelectrical transduction by sensory hair cells.

### 2.1. The Critical Role of the Stria Vascularis in Maintaining Cochlear Electrochemical Homeostasis

The stria vascularis is one of the most metabolically active structures in the cochlea [11]. This highly vascularised tissue ensures a constant supply of oxygen and nutrients to the inner ear to meet its substantial energy demands. The stria vascularis consists of three main cell types: marginal cells (facing the endolymph), intermediate cells, and basal cells (facing the spiral ligament) (Figure 1C). Tight junction proteins within the marginal and basal cells of the stria vascularis are essential for maintaining the separation of endolymph from perilymph and preserving the distinct ionic environment in the stria vascularis required for K^+^ secretion [12].

Stria vascularis generates a small positive charge (the EP) in the endolymph by secreting K^+^ against a steep concentration gradient. This electrical potential is measured in the order of +80–100 mV [6], and combined with the polarised hair cells, results in the rapid influx of K^+^ into hair cells during sensory transduction [3,13]. This specialised transduction is initiated by the hair cell mechanoelectrical transduction (MET) channels, which open when stereocilia are deflected by sound-induced fluid vibrations. This facilitates a rapid influx of K^+^ ions, depolarising the hair cells, opening of voltage-gated Ca^2+^ channels, and triggering Ca^2+^-dependent neurotransmitter (glutamate) release into the synaptic cleft. This electrical activity initiates action potentials in the spiral ganglion neurons, the primary afferent neurons in the cochlea [14]. The generation of the EP by the stria vascularis incurs a high metabolic cost. Consequently, any compromise in strial function, such as vascular insufficiency or metabolic dysregulation, can lead to a collapse of the electrochemical gradients critical for sensory transduction [15]. Age-related changes in strial metabolism associated with reduced vascularisation, chronic inflammation, and mitochondrial DNA damage can be important contributors to SNHL in older populations [11,16].

### 2.2. Ion Transport and K^+^ Recycling

The maintenance of a K^+^ gradient is a cornerstone of cochlear homeostasis [3]. Once K^+^ ions enter the hair cells through MET channels, they must be efficiently recycled to sustain the EP and prevent their toxic accumulation around the basolateral surfaces of sensory and supporting cells. To enable this, the supporting cells in the organ of Corti, the fibrocytes in the spiral ligament and spiral limbus, the mesenchymal cells lining the scala vestibuli and the marginal and intermediate cells of the stria vascularis form an extensive intercellular communication network mediated by gap junctions. This network, comprising connexin hemichannels such as connexin 26 and connexin 30, creates a system for the rapid ion transport between cells, which ultimately facilitates a K^+^ recycling loop that redistributes the K^+^ ions back to the stria vascularis [17].

Ion pumps and channels located in the stria vascularis complement the function of these gap junctions in the K^+^ recycling loop (Figure 1C). Intermediate cells express inwardly rectifying K^+^ channels, such as Kir4.1 (KCNJ10) [18,19]. The Kir4.1 channels are critical for the EP generation because they allow K^+^ to diffuse from intermediate cells into the intrastrial space. The Na^+^/K^+^-ATPase pump on the basolateral side of marginal cells, which uses intracellular ATP to drive the active transport of ions, is crucial for maintaining the high K^+^ concentration inside the marginal cells of the stria vascularis [3]. The Na^+^/K^+^/2Cl^−^ cotransporter (NKCC1) facilitates the further uptake of ions from the intrastrial space, consolidating the buildup of K^+^ in marginal cells [20]. Potassium KCNQ1/KCNE1 channels on the apical membranes of marginal cells assist in establishing a pathway for K^+^ diffusion from marginal cells to endolymph [3]. Mutations in either of these two subunits cause Jervell–Lange–Nielsen syndrome in humans [21] characterised by hearing loss and cardiac arrhythmia. Precise regulation of these ion channels and transporters, along with efficient transport by gap junctions, ensures that the K^+^ concentration gradient generated by active ion transport is maintained, which is an integral requirement for sensory transduction.

## 3. Adaptive Mechanisms in the Cochlea to Maintain Homeostasis

When exposed to cellular stress, the cochlea mounts compensatory responses aimed at restoring homeostasis. Upregulation of protective proteins, increased expression of antioxidant enzymes, and the activation of signalling pathways that promote cell survival are all part of the defence mechanism [22,23]. However, these compensatory mechanisms have limited capacity. When the intensity or duration of the insult exceeds these protective limits, compensatory responses become overwhelmed, leading to irreversible cellular damage. For example, long-lasting or repeated noise exposure progressively diminishes the cochlea’s capacity for repair, eventually culminating in widespread hair cell loss and permanent sensorineural hearing impairment [24].

### 3.1. Adaptive Mechanisms Against Oxidative Stress

One of the leading causes of the disruption of cochlear homeostasis is oxidative stress. Noise trauma, ageing, and ototoxic drugs are all known to generate elevated levels of reactive oxygen species (ROS), which can damage cellular membranes, proteins, and DNA in the cochlea [25]. Tissues with high metabolic demands, such as the stria vascularis and the outer hair cells, are particularly vulnerable to the damaging effects of ROS-induced oxidative stress [26]. Excessive production of mitochondrial ROS causes oxidative damage to key mitochondrial components, such as mitochondrial DNA (mtDNA), mitochondrial membranes, and respiratory chain proteins, resulting in mtDNA mutations, lipid peroxidation, and protein oxidation [26,27]. The resultant oxidative damage can compromise the ion channels and pumps critical for maintaining the ion composition in endolymph, as previously demonstrated in the ageing brain [28]. In addition, excessive ROS production leads to calcium influx into sensory hair cells, which in turn causes a surplus of glutamate release and excitotoxicity [29].

Interestingly, there are also intrinsic protective mechanisms at play in the cochlea. The expression levels of several endogenous antioxidant enzymes (e.g., glutathione peroxidases and reductase, superoxide dismutase, catalase) increase in response to metabolic stress, noise exposure, or ototoxic drugs [30]. These enzymes can attenuate the damaging effects of ROS and mitigate oxidative stress, a major cause of hair cell death.

### 3.2. Signalling Pathways Activated by Stress and Injury in the Cochlea

Specialised regulatory proteins and signal transduction pathways within the cochlea collaborate to sustain cellular viability and functionality. Mitogen-activated protein kinase (MAPK), phosphoinositide-3 kinase/protein kinase B (PI3K/Akt), calcium channels, and ROS signalling pathways regulate the development and survival of auditory hair cells in response to environmental and metabolic challenges [22]. Dysregulation in these signalling pathways can tip the balance from cell repair to cell death, contributing to SNHL [22,31].

Another receptor-mediated signalling pathway activated under stress is purinergic signalling via ATP-gated ion channels (P2X receptors) [32]. Exposure to moderate noise levels, causing a temporary threshold shift, elevates extracellular ATP in the endolymph and activates P2X2 receptors in the cochlear partition. The activation of P2X2 receptors provides a K^+^ shunt conductance away from the endolymphatic compartment, which reduces the driving force for sound transduction and, consequently, hearing sensitivity in mice [33]. This mechanism may contribute to protective hearing adaptation with sustained elevated sound levels that can cause permanent hearing loss [5].

Adenosine receptor signalling is also a potent regulator of cochlear response to stress. Activation of adenosine A_1_ receptors can mitigate cochlear injury caused by acoustic overexposure and ototoxic drugs (cisplatin and aminoglycosides) [32,34]. Similarly, inhibition of adenosine A_2A_ receptors can mitigate excitotoxic injury in organotypic tissue culture of the rat cochlea [35] and delay the progression of age-related hearing loss in mice [36]. The balance between A_1_ and A_2A_ receptors appears critical for cochlear response to stress and injury [32].

Furthermore, key growth factors, including brain-derived neurotrophic factor (BDNF) and neurotrophin-3 (NT-3), released from sensory and supporting cells of the organ of Corti, are involved in cellular repair processes within the cochlea [2]. Administration of exogenous neurotrophins promotes synaptic regeneration of the ribbon synapses in sensory hair cells and enables hearing rescue in mice and guinea pigs following acoustic trauma [37,38]

### 3.3. Epigenetic Modifications and Homeostatic Regulation by MicroRNAs

Recent studies suggest that epigenetic factors, such as DNA methylation and histone modification, might play a crucial role in determining the susceptibility of cochlear cells to oxidative and metabolic stress [39]. These epigenetic modifications can influence the differentiation, development, and protection of sensory hair cells in the cochlea, potentially leading to hair cell degeneration and hearing loss [40,41]. Manipulating the epigenetic status could be a tool to regulate the expression levels of critical protective proteins and ion channels, and may even alter the limited regenerative capacity of cochlear cells [39,41].

Other studies have shown that microRNAs (miRNAs), a class of short non-coding RNAs that regulate the expression of mRNA and protein targets, are important regulators of cellular senescence and ageing [42]. These studies have raised the possibility that miRNAs are responsible for fine-tuning the expression of genes involved in protective and degenerative processes in the cochlea [43]. The identification of hundreds of miRNAs in the auditory system, along with a better understanding of the functions of many of these miRNAs, holds promise for their use as inner ear therapeutics [44,45,46].

### 3.4. The Cochlear Microenvironment, Intercellular Communication and Systemic Health

The cochlea is not an isolated system; it operates in concert with systemic physiological processes such as blood flow, immune regulation, and systemic metabolism. Intact and healthy vasculature and blood flow are crucial for cochlear homeostasis [47]. Mounting evidence suggests that disruption to the blood-labyrinth barrier (BLB) function leads to hearing loss in models of ototoxic [48], genetic [49], and systemic diseases [50]. Increased attention is now being focused on understanding how systemic factors, such as blood circulation, influence BLB and cochlear homeostasis. For instance, conditions like diabetes and hypertension are known to impair vascular supply and thereby disrupt the function of the stria vascularis [51]. In addition, there is strong evidence that the cochlea is vulnerable to systemic inflammation [52]. Such biomedical evidence of increased prevalence of hearing loss associated with poor health (e.g., diabetes-related) aligns with epidemiological observations in the human population [53]. Therefore, therapeutic strategies that improve systemic health may have a beneficial impact on cochlear homeostasis and resilience to stress.

Intercellular communication via extracellular vesicles, such as exosomes, is another potential area of investigation. In other systems, these vesicles mediate communication between cells by transferring small RNAs, proteins, and other bioactive molecules [54]. In the cochlea, heat stress stimulates exosome release, and these vesicles promote the survival of hair cells under ototoxic stress [55]. The exosome signalling between hair cells, supporting cells, and immune cells may constitute a vital component of the adaptive response to maintain the homeostatic regulatory network in the cochlea [56]. A better understanding of the exosome vesicular transport in the cochlea may lead to the use of exosomes containing therapeutic molecules (e.g., miRNA-21) [57]. Research focusing on the cellular distribution and trafficking of exosome vesicles could eventually lead to innovative biological therapies to restore cellular function in the cochlea.

## 4. Sensorineural Hearing Loss: Causes and Cellular Impact

SNHL is the most common form of permanent hearing loss arising from the loss of sensory hair cells, synaptic connections between sensory hair cells and the afferent terminals of the spiral ganglion neurons, with ultimate loss of primary auditory neurons in the spiral ganglion [58]. Dysfunction of the central auditory pathways can also play an independent role in SNHL [31]. SNHL manifests clinically as muffled hearing, reduced perception of high-pitched sounds, and difficulties with speech discrimination, particularly in noisy environments [59]. The deterioration in auditory perception is typically irreversible because the sensory hair cells of the cochlea have a very limited capacity for regeneration in mammals. Hearing loss can range from mild to profound and is often accompanied by tinnitus, the perception of phantom sounds, further disrupting the quality of life for affected individuals.

**Figure 2:**
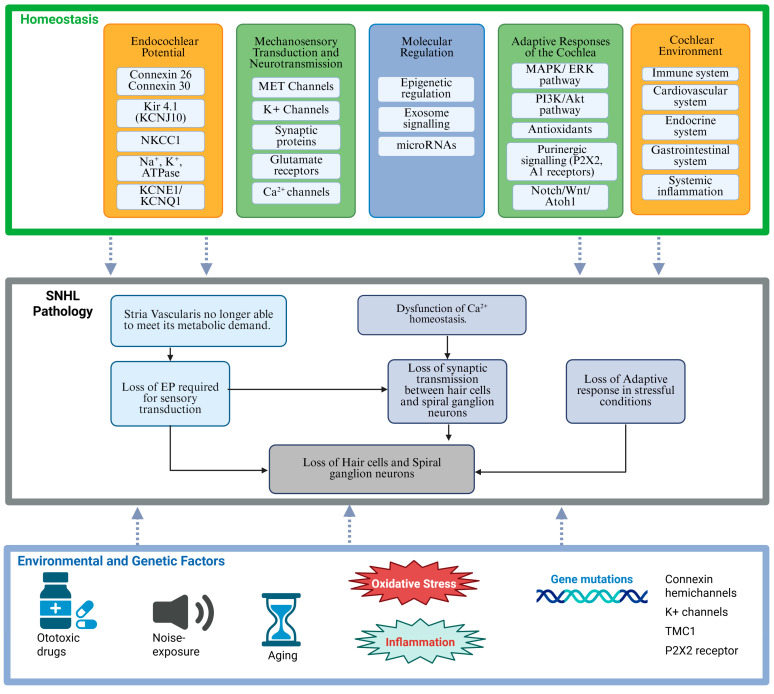
Sensorineural pathology and hearing loss resulting from dysfunctional cochlear homeostasis. This schematic diagram illustrates the essential elements of normal cochlear homeostasis (upper panel) and the environmental and genetic factors that can overwhelm adaptive responses in the cochlea (bottom panel), ultimately leading to SNHL pathology and hearing loss (middle panel). Created in BioRender. Suzuki-Kerr, H. (2025) https://BioRender.com/zn7lur4 (accessed on 2 November 2025).

Multiple factors contribute to the onset of SNHL (Figure 2). Genetic mutations affecting connexin proteins, ion channel function, or other critical regulatory molecules can predispose individuals to early-onset SNHL [60]. Age-related hearing loss is linked to genetic risk factors but is also influenced by a lifelong cumulative effect of environmental stressors, such as noise, otological diseases, or ototoxic drugs [16]. Acquired forms of SNHL may result from chronic noise exposure, common in industrialised and some recreational environments. Ototoxic medications, particularly aminoglycoside antibiotics and chemotherapeutic agents such as cisplatin, can also induce hair cell damage by directly activating intracellular pathways leading to apoptosis [61,62]. New evidence suggests that macrophage-induced inflammation also contributes to cellular damage following cisplatin treatment [63]. Systemic conditions such as diabetes, hypertension, and mitochondrial disorders have been linked to vascular or metabolic disruptions within the inner ear [64,65], further highlighting the critical role of cochlear homeostasis in the maintenance of normal auditory function.

### 4.1. Cellular Impact of Disruption in Homeostasis in Sensorineural Hearing Loss

Understanding how cochlear homeostasis is disrupted by environmental, metabolic or genetic factors provides critical insights into the pathophysiology of SNHL. When the cochlear homeostasis is disturbed, the resulting cascade of events precipitates cellular injury and permanent loss of function. The sensory hair cells, supporting cells, and secretory epithelial cells of the stria vascularis are extremely sensitive to disturbances in their microenvironment [66,67]. Numerous experimental models have highlighted the link between cochlear disorders and impaired homeostatic mechanisms. For example, in studies where the expression of gap junction proteins was genetically disrupted, a concomitant decrease in K^+^ recycling efficiency was observed [68]. In parallel, animal models subjected to oxidative stress exhibited significant dysregulation of ion pumps and various signalling pathways [69], further highlighting the vulnerability of cochlear homeostasis in the face of environmental and metabolic challenges.

### 4.2. Metabolic Stress, Oxidative Damage, and Apoptosis

The cochlea is particularly vulnerable to energy deficits and oxidative stress due to its high metabolic demands. For instance, the temporary loss of blood supply to the cochlea may underlie the sudden onset SNHL due to energy deficits [70]. Reduced vascularisation in the cochlea, cumulative oxidative stress, and low-grade cochlear inflammation play critical roles in the development of age-related hearing loss [71]. Noise exposure, ischemia, and ototoxic drugs can all dramatically increase ROS production, overwhelming the cochlear antioxidant defences [72]. Oxidative stress can undermine the functional integrity of membrane proteins vital for ion transport and signal transduction [4]. These insults trigger a cascade of intracellular events that can lead to cell death through apoptosis or necrosis. Once hair cells are lost, the damage is irreversible, leading to permanent SNHL. Furthermore, damage to supporting cells can hinder the essential recycling of ions necessary for maintaining the EP, thereby amplifying the degenerative process [73].

### 4.3. Immune Activation and Inflammation in the Cochlea

Inflammatory processes represent another critical component leading to the breakdown of cochlear homeostasis. Following cochlear injury caused by noise, drugs, or other insults, immune mediators, such as cytokines and chemokines, are released locally from infiltrating immune cells, including macrophages [74,75,76]. Macrophages are the primary innate immune cells in the cochlea, playing crucial roles in cochlear immune homeostasis, repair, and pathology [77]. There are several macrophage phenotypes present in the human cochlea from early inner ear development through to adulthood [78,79], where they likely play roles in cochlear homeostasis, trophic support and patterning, in addition to their well-characterised immune functions [79]. The cochlea contains resident macrophages in various cochlear regions, such as the basilar membrane, osseous spiral lamina, spiral ganglion, spiral ligament, and stria vascularis [80,81]. The inflammatory response involves the release and upregulation of inflammatory molecules from macrophages and other immune cells, including cytokines like IL-1β, TNF-α, TGF-β, and IL-10, chemokines like CX3CR1 and adhesion molecules (ICAM-1) [82,83]. Cytokines are signalling molecules that influence the activity of other immune cells and can either promote or resolve inflammation, whereas chemokines attract immune cells to the site of injury or inflammation [84]. While these inflammatory responses may initially function in a protective capacity by mobilising repair mechanisms, chronic inflammation can exacerbate cellular dysfunction and damage and accelerate hearing deterioration [82,85]. The chronic activation of inflammatory cascades has been implicated in animal models of noise-induced, drug-induced, and age-related hearing loss [16,62,63,84]. Control of such inflammatory responses is emerging as a promising target for therapeutic interventions [84,85].

### 4.4. The Interplay Between Genetic and Environmental Factors

While environmental factors, such as noise or exposure to ototoxic compounds, play a significant role in disrupting cochlear homeostasis, genetic predispositions can increase vulnerability to damage. Of over 150 genes identified to be associated with human SNHL, mutations in genes encoding ion channels (e.g., *KCNQ1*, *KCNQ4*, *TMC1*, *P2RX2*, *KCNJ10*) [86,87,88,89], connexin hemichannels (e.g., *GJB2*, *GJB6*) [90,91] or synaptic proteins (*OTOF*) [92] have also been associated with hereditary forms of SNHL. In individuals harbouring such mutations, even minor environmental insults may lead to a disproportionate collapse of cochlear homeostasis. For example, a mild genetic defect in ion channel function may maintain near-normal hearing in an affected individual until a significant noise insult pushes the system beyond its compensatory capacity [93]. The disruption of cochlear homeostasis may thus lead to permanent damage of sensorineural tissues due to the combined effects of multiple environmental and genetic factors (Figure 2).

## 5. Current Research and Therapeutic Approaches

In response to the growing burden of SNHL, it is more important than ever to advance therapeutic strategies that target the fundamental cause of failed cochlear homeostasis, rather than treating the symptoms of dysfunction. The complexity of the cochlear microenvironment and the heterogeneous pathogenesis of sensorineural cochlear damage have prompted a range of innovative strategies aimed at maintaining cochlear homeostasis. These range from pharmacological interventions to gene therapy, cellular regeneration, cell replacement, and advanced biomolecular and bioengineering approaches.

### 5.1. Antioxidant and Anti-Inflammatory Therapies

Given the central role of oxidative stress in disrupting cochlear homeostasis, pharmacological strategies targeting ROS and promoting mitochondrial health have gained momentum. Antioxidants have been investigated for their ability to mitigate noise-, drug and age-related oxidative damage [72,94,95]. Compounds that support mitochondrial function have also been tested in preclinical models with encouraging outcomes [96,97]. By stabilising mitochondrial membranes and reducing the production of ROS, these agents help preserve the intricate balance of ion transport and metabolic activity within the cochlea.

Inhibition of specific inflammatory mediators is also being explored to mitigate the adverse effects of chronic inflammation in the cochlea [84,98]. Several clinical trials assessed whether antioxidant and anti-inflammatory interventions can protect at-risk populations from progressive hearing loss, such as individuals chronically exposed to noise or those undergoing cisplatin chemotherapy [99]. For example, the antioxidant sodium thiosulfate (STS) has been clinically approved to protect paediatric patients from cisplatin-induced hearing loss [100]. While preclinical and limited clinical data suggest STS may also be effective in adults, more studies are needed to confirm its safety and efficacy in the adult population [101]. This underscores the need to understand the entire range of perturbations to cochlear homeostasis in order to triage and stratify patient populations into trials that are most likely to result in improved function.

### 5.2. Gene Therapy to Restore Molecular Equilibrium

Gene therapy holds promise for correcting the genetic defects that underlie many forms of congenital SNHL, where the causal genes have been identified [102]. Viral vectors such as adeno-associated viruses (AAV) are being optimised for delivery to the cochlea, targeting specific cell types [103]. Therapeutic vectors can be delivered directly into the cochlear fluid space, allowing for even distribution in animal models [103]. Innovative delivery approaches continue to be developed, including specialised dual-lumen microneedles that simultaneously inject viral vectors while removing the same volume of perilymph [104]. Such solutions confer significant benefits over routine intratympanic injections by facilitating the delivery of larger volumes of therapeutic agents to the inner ear, whilst minimising disruption to the intracochlear environment.

There are three main types of viral vector-based gene therapy: gene replacement (including gene augmentation), gene editing and gene silencing [102,105]. Gene replacement therapy aims to deliver a functional gene to diseased or dysfunctional cells, essentially “correcting” the genetic defect. Gene augmentation introduces a functional copy of a gene into cells to compensate for a mutated or missing gene in diseases caused by loss-of-function mutations. Delivering functional copies of genes encoding critical structures in the cochlea, such as connexin hemichannels or ion channel subunits, can potentially restore the homeostatic mechanisms required for hearing [102]. Historically, these techniques have been limited by the packing size of AAVs (~4.8 kb); however, advances in dual AAV strategies have allowed for the longer sequence of many deafness genes to be successfully delivered in vivo [105,106]

Gene editing enables the precise modification of genetic material in situ, often utilising the CRISPR-Cas9 technology to remove a defective sequence while preserving normal expression patterns [105]. In contrast, gene silencing inhibits the translation of a disease-causing gene into a protein/s, for example, using antisense oligonucleotides, small interfering RNAs (siRNAs) or microRNAs to stop abnormal protein production [105]. The type of gene therapy selected depends on the underlying pathology and the extent to which residual cochlear cytoarchitecture is available for reprogramming. This was demonstrated in early gene therapy studies, which clearly demonstrated the need for residual cells/structures for adequate expression of introduced genes [107,108].

The most popular gene therapy approaches for inner ear restoration thus far fall into the gene replacement category. In breakthrough clinical research, hearing recovery was reported in both a unilateral [106] and bilateral [109] gene therapy clinical trials, treating children with autosomal recessive deafness 9 (an otoferlin gene mutation). Results of these trials demonstrated both the safety and efficacy of the treatment, as measured by significant improvements in hearing thresholds, speech perception, and sound source localisation [106,109].

Additional gene therapy trials in the pipeline aim to restore MET channel function in hair cells, mutations which cause permanent SNHL [110,111]. Promising preclinical work has demonstrated the recovery of normal hearing function by multiple objective measures following a single injection of gene therapy via AAV targeted to the inner ear. Moreover, preclinical studies [112,113,114] have demonstrated that gene therapy can partially restore ion channel or glutamate transport function in the cochlea. Some of these studies utilised murine models of Jervell–Lange–Nielsen syndrome and a type of deafness caused by a deficiency in vesicular glutamate transporter-3 (VGLUT3). Furthermore, gene therapy strategies are being developed to mediate protective responses in the context of ototoxic stress [115,116].

Fewer studies have aimed for gene augmentation or direct reprogramming for inner ear repair. However, these strategies may hold value when more extensive damage has occurred. A large part of the success of early gene therapies is likely due to the presence of residual structures in the organ of Corti. For instance, in otoferlin gene therapy trials, both the hair cells and auditory neurons remain present, at least for several years after birth.

These landmark studies mark the beginning of a new era in cochlear therapeutics. The successful delivery of new genes has been dramatically improved using cochlear perfusion [117]. Although the safety and efficacy of gene therapies have been demonstrated in the short term, the major challenges remaining include the longevity of the therapy, achieving robust results across different gene mutations, and ensuring that the host immune response does not interfere with subsequent gene delivery [118]. Advances in the design of tailored viral vectors, including dual vector approaches as demonstrated in successful otoferlin trials [106], and the exploration of non-viral delivery systems, such as nanoparticles, are promising paths that may further improve these therapies. This includes ongoing attempts to regenerate hair cells following their destruction, a therapy that holds considerable potential for reversing damage for the much larger numbers of patients experiencing SNHL [118].

### 5.3. Regenerative Medicine and Stem Cell Therapies

Perhaps the most transformative approach for SNHL is the prospect of regenerative medicine. The cells of the inner ear, including both the sensory hair cells and neurons, are known for their limited regenerative capacity. Thus, advances in stem cell biology offer hope for their replacement after irreversible damage or death of these cells. Early investigations into stem cell therapy for hearing loss demonstrated the feasibility of engrafting stem cells into the mammalian cochlea to replace either sensory hair cells [119] or spiral ganglion neurons [120,121]. Subsequent transplantation studies have reported the successful integration of human stem cell-derived auditory neural progenitors into various animal models, including mice [122,123], gerbils [124], and guinea pigs [125]. Building on this pre-clinical progress, these former “blue sky” approaches have received regulatory approval, with the UK-based company Rinri Therapeutics poised to commence first-in-human stem cell transplants designed to regenerate damaged auditory neurons [126]. Patients in the trial have severe-to-profound SNHL and will receive both a cochlear implant and a cell transplant. Cochlear implants are the standard treatment option for these patients; therefore, the first trials aim to demonstrate safety.

Beyond transplantation, stem cells are also being used to generate in vitro tissue models. These models hold promises for a better understanding of disease, correcting gene mutations, and as platforms for drug screening, prior to advancement into preclinical testing [127,128,129]. The most advanced of these in vitro models are inner ear organoids: three-dimensional, simplified and miniaturised versions of an organ that are grown using stem cells [127]. Organoids comprise multiple cell types, arranged in a manner that partially mimics the structure and function of the corresponding real organ. This arrangement enables the replication of certain aspects of development, physiology, disease, and regenerative processes. Inner ear organoids have advanced significantly in recent years, with a landmark paper illustrating their similarity to human inner ear tissues using single-cell transcriptomics [127]. Interestingly, these studies confirmed a preference for producing vestibular hair cells in inner ear organoids [127], supporting earlier organoid studies drawing the same conclusions [128].

Regenerative approaches have also explored the activation of latent supporting cells within the cochlea [130]. The re-expression of transcription factors ATOH1, GFI1, and POU4F3 in the mature cochlea reprograms non-sensory cells into hair cells [131,132]. ATOH1 acts as a master switch, further guiding these progenitors towards a hair cell identity. In non-mammalian vertebrates, such as birds, fish and amphibians, supporting cells can transdifferentiate into hair cells following injury [133,134]. Unlocking the molecular switches that permit such plasticity in the mammalian cochlea is an active area of research. Small molecules and gene editing techniques that modulate pathways such as Notch and Wnt/β-catenin are at the forefront of attempts to induce cochlear cells back into a regenerative state [135]. These attempts include more than just the hair cells, with advances made in the direct reprogramming of intracochlear non-neuronal cells into auditory neurons, achieved by overexpressing two transcription factors (ASCL1 and NEUROD1) [136]. The direct reprogramming of non-neuronal cells into new auditory neurons may prove useful in pathological conditions where the hair cells remain functional, but the neurons are dysfunctional or missing (e.g., auditory neuropathy or hidden hearing loss).

### 5.4. Bioengineering Innovations

Bioengineering innovations are poised to offer complementary approaches to restore cochlear homeostasis. Advanced biomaterials, for example, are being developed to provide scaffolding for cell transplantation, ensuring proper alignment, survival, and integration of new cells [137,138]. Moreover, the use of nanotechnology to deliver ions, growth factors, and/or pharmacological agents directly to the inner ear represents an alternative strategy to enhance cochlear protection from stress and injury [139].

Electrical stimulation devices, such as cochlear implants, have revolutionised the treatment for profound hearing loss. However, these devices do not restore natural cochlear homeostasis, and ongoing research aims to combine implant technology with regenerative approaches. This is evidenced by hybrid drug-eluting cochlear implant devices that integrate biomolecules with electronic functionality, which may eventually bridge the gap between prosthesis and organic repair, providing a natural and dynamic restoration of hearing [140,141].

In addition, clinical trials are underway that leverage the stimulating parameters of a cochlear implant to deliver growth factors to the inner ear. These growth factors support the long-term survival of auditory neurons and encourage their peripheral dendrites to grow towards the stimulating electrode. The trial, known as the BaDGE^®^ platform, is led by the University of New South Wales [142] and involves a novel proprietary technology that enables precision delivery of DNA/RNA therapeutics into the cochlea using an innovative pulsed electric lensing technology. The new DNA/RNA is delivered to the cochlea during cochlear implant surgery, driving directed regeneration of the auditory nerve fibres and ‘closing the neural gap’ between the nerve fibres and the electrode, thus improving device performance [143].

### 5.5. Emerging Biomarkers and Personalised Medicine

One of the essential requirements for developing advanced biomolecular therapies for SNHL is the early identification of homeostatic disruption. Advances in molecular diagnostics have led to the discovery of several biomarkers, ranging from genetic polymorphisms and epigenetic signatures to proteomic profiles associated with oxidative stress or inflammation [144]. For instance, increased levels of C-reactive protein (CRP) and IL-6 were found in blood samples of individuals with age-related hearing loss [145], while a voltage-sensitive motor protein, prestin, is the main contender as a biomarker of outer hair cell damage [146]. Circulating microRNAs are excellent candidates for the early detection of inner ear disease, as they are cell-independent and stable in many biological fluids [147]. These biomarkers have the potential to identify individuals at increased risk of hearing loss before irreversible damage occurs, thereby opening a therapeutic window for early intervention. They may also be used to accurately assess the site of damage in the inner ear, thus improving patient management and treatment efficacy.

Personalised medicine approaches are particularly important to develop, given the heterogeneity of SNHL aetiologies. Next-generation sequencing is increasingly favoured for genetic diagnosis due to its capacity for large-scale genetic screening to identify gene variants that increase the risk of SNHL over time [148]. By tailoring interventions to the specific genetic and health profiles of patients, clinicians are likely to be able to optimise therapeutic outcomes. Other examples include the use of ultrasharp microneedles for the safe sampling of inner ear fluids [149] and the local delivery of gene therapy using nanoparticle vectors [150]. Either through gene therapy, targeted pharmacologic strategies, regenerative medicine, or a combination of these techniques, the future of SNHL treatment ultimately rests in our ability to precisely detect and correct disturbances in cochlear homeostasis at an early stage [150].

### 5.6. Limitations and Barriers to the Uptake of Emerging Treatments for Hearing Loss

There are numerous limitations for the therapeutic application of these emerging technologies which need to be addressed prior to their widespread use. These limitations are summarised in Table 1, and include development of more sensitive diagnostics, optimisation of delivery approaches, measuring the longevity of new approaches, screening and optimal patient selection, cost and safety. Whilst rapid progress is being made toward inner ear gene therapy approaches for specific mutations such as otoferlin [106,109], broad, robust, and safe regenerative treatments for human hearing loss remain a significant challenge for many reasons.

Now that the feasibility of these approaches has been established, or at the very least is well underway, attention ought to be focussed on diagnostics. Early identification of alterations to inner ear homeostasis could streamline faster treatments, resulting in more effective and less costly outcomes than waiting for potentially more severe and permanent damage to occur. This requires precision diagnostics that can stratify patient populations and pinpoint the site(s) of damage. Improved diagnostics could also provide patients with a variety of treatment options currently lacking, leading to better care, improved hearing, and enhanced health and well-being. This area is currently underrepresented and presents opportunities for development.

A key limitation of cell and gene therapy for inner ear disease is the robust delivery of healthy cells/genes/drugs to the correct anatomical targets. This has never been a simple matter of injection, given the complexity of inner ear biology, which involves delivering drugs through the bone to thin membranes within a fluid-filled chamber, and/or attempting to achieve effective drug concentrations through systemic application. After decades of research, several key advances have emerged, including the use of a second inner ear fenestration for better cochlear perfusion of genes (currently approved for otoferlin gene therapy [106,109]) and direct injection techniques into the VIII cranial nerve for imminent stem cell transplantation therapy [151]. Emerging fluid-based delivery techniques include specialised dual micro-needles [104], which circumvent the need for inner ear fenestrations, thereby preserving more of the inner ear cytoarchitecture during the surgical process.

While initial gene therapy trials are cause for optimism, there remains a need to measure improvement over the longer term to ensure that treatments are long-lasting. Similarly, we do not yet know how long stem cell transplants will survive in vivo, nor whether they will form functional connections with their appropriate cell targets [152]. The field eagerly anticipates the results from imminent Phase I/II first-in-human trials and will be guided by new technologies, such as “cell cloaking,” to avoid immune rejection [153], as well as ongoing results from related studies in the nervous system aimed at treating Parkinson’s disease [154].

Like all new technologies, there will be a cost, which will necessarily include genetic screening, careful growth of Good Manufacturing Practices (GMP)-grade cells for transplantation, surgeries, imaging and audiometry to ensure both safety and efficacy in the long term. There are also ethical questions surrounding the source of stem cells, the viral vectors used, and equitable access to these new therapeutics for all.

## 6. Integrative Perspectives on Cochlear Homeostasis and Hearing Loss

The active transport of ions, sound transduction by hair cells, and the ongoing metabolic support provided by the stria vascularis represent a fine balance that is susceptible to both acute and chronic perturbations [155]. This equilibrium is dynamic, continually adjusting in response to environmental challenges, metabolic demands, and age-associated changes. The cochlear capacity to adapt to these changes through regulatory proteins, gap junctions, and repair mechanisms is a tribute to its evolutionary design. However, when the challenges exceed the homeostatic capacity, a loss of function can occur immediately, and this is often irreversible.

The existing literature suggests that diverse insults converge on a limited set of homeostatic systems in the inner ear, including ion balance, redox status, vascular supply, fluid volume regulation, immune and metabolic status. Understanding hearing loss thus requires viewing genetics, environmental factors, ageing, and inflammation not as isolated causes but as perturbations of a cochlear ecosystem. In many cases, it is the cumulative and/or combined effect of several stressors that precipitates the irreversible damage or loss of the critical cells in the cochlea.

**Figure 3:**
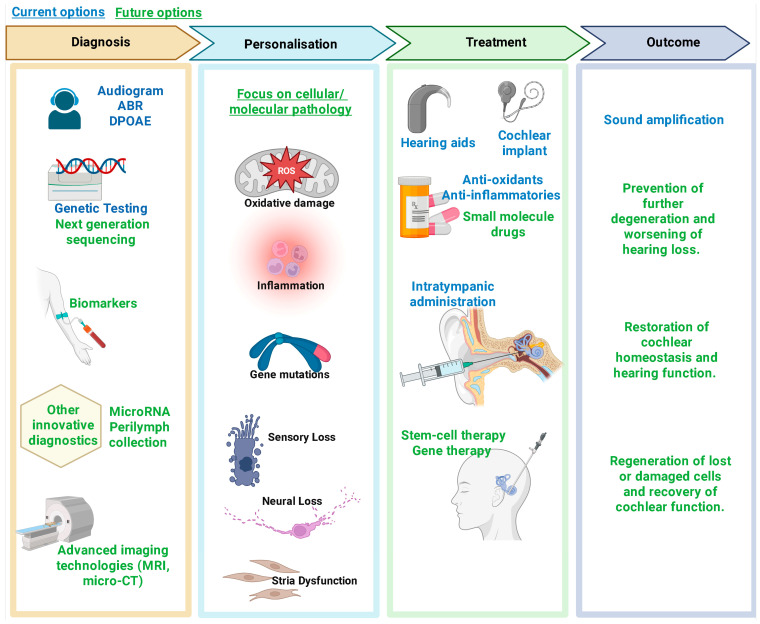
High-level overview of diagnosis and treatment of hearing loss, focusing on cellular and molecular pathology. The growing fields of pharmacological, gene and stem cell therapies, including regenerative medicine, have begun to offer the promise of permanent hearing restoration. ABR, auditory brainstem responses; DPOAE, distortion product otoacoustic emissions. Created in BioRender. Suzuki-Kerr, H. (2025) https://BioRender.com/tx6blis (accessed on 2 November 2025).

An excellent example of a multifactorial inner ear disorder caused by the disruption of cochlear homeostasis is Ménière’s disease (MD). In MD, cochlear homeostasis is primarily disrupted by an abnormal accumulation of endolymph in the inner ear (endolymphatic hydrops). This fluid imbalance alters ion regulation, damages the blood–labyrinth barrier, and disrupts cochlear mechanics, leading to vertigo, tinnitus, and fluctuating hearing loss [156]. Current models frame MD as a failure of the inner ear homeostatic network, particularly the endolymphatic sac, which normally provides pressure relief and long-term volume regulation. Primary or secondary dysfunction of the sac, combined with additional stressors (immune, vascular, hormonal, or osmotic), can overwhelm compensatory mechanisms and precipitate persistent endolymphatic hydrops, cellular damage, and cochlear homeostatic breakdown [157,158]. Multifactorial vulnerability leading to hearing loss in MD thus necessitates a comprehensive therapeutic strategy that addresses both cellular injury and the underlying disturbances in cochlear homeostasis. The same strategy applies essentially to all hearing disorders (Figure 3).

## 7. Concluding Remarks and Future Directions

Translating basic research on cochlear homeostasis into clinical therapies has made significant strides in recent years. Pre-clinical animal models have accelerated our understanding of the complexities of ion channel function, mitochondrial resilience, and regenerative potential in the inner ear. The advent of gene therapy, stem cell transplantation, and nanodelivery systems has offered substantial opportunities for clinical intervention, and both stem cell and gene therapies have now progressed to Phase I/II clinical trials. Future research is poised to address several key areas:

- Improved Delivery Systems

Continued efforts in bioengineering, such as the development of nanoparticle carriers and improvements to viral vectors, are expected to enhance the effectiveness, specificity, and safety of gene and drug delivery in the cochlea. In addition to refinements in surgical approach and the use of microneedles, these innovations will be essential for delivering therapeutics to the exact regions where homeostatic imbalance occurs.

- Identification of Molecular Targets

High-throughput screening, such as next-generation sequencing and advanced imaging technologies, facilitates the discovery of new molecular targets involved in ion transport and the regulation of oxidative stress and inflammation. With the identification of novel targets, drugs can be designed to modulate their activity, providing another way to restore or preserve cochlear homeostasis. Similarly, new MRI techniques have been developed to quantify white matter fibre pathways in individuals with normal and abnormal hearing [159,160], and these may provide additional objective measures for emerging cell and gene therapies.

- Regenerative Medicine

Unlocking the regenerative potential of the cochlea remains one of the most exciting research avenues. Regenerative medicine may one day offer a permanent cure for SNHL, either through the reprogramming of supporting cells, the use of stem cell transplants, or the activation of latent regenerative pathways. A key focus is on ensuring that newly generated cells form synaptic connections with their targets, to re-establish the sensorineural circuitries essential for hearing.

- The Role of Inflammation and Immune Modulation

Understanding the dual roles of inflammation, both as a repair mechanism in the cochlea and as a propagator of cell damage, will be necessary to design interventions that can modulate immune responses at the right time. Selective anti-inflammatory agents that preserve protective immune functions while suppressing chronic inflammation would be highly effective.

- Personalised Therapeutic Strategies

With advances in genomics and molecular diagnostics, the future of SNHL treatment is likely in personalised medicine. By adapting interventions to the genetic and environmental profiles of individual patients, clinicians can offer tailored therapies that target the precise disruptions in cochlear homeostasis before permanent damage occurs.

In summary, the maintenance of cochlear homeostasis is crucial for preserving the sensorineural function of the inner ear. Disruption of this exquisite balance through genetic, environmental, or metabolic alterations sets the stage for irreversible SNHL. While current treatments for SNHL are largely palliative, the growing fields of gene and pharmacological therapies, as well as regenerative medicine, have begun to offer the promise of permanent hearing restoration. The multifaceted nature of cochlear homeostasis necessitates a comprehensive approach that not only addresses the symptoms of hearing loss but also targets the root causes at the cellular and molecular levels.

## Figures and Tables

**Table 1:** Challenges for novel hearing loss treatments.

	Stem Cell Therapies	Gene Therapies	Drug Therapies
**Delivery challenges**	Direct delivery approaches that preserve cochlear tissue and limit migration beyond target regions	Variable delivery of viral vectors to target cells; constraints in delivery via inner ear fluids	Variable drug concentration and distribution, depending on the delivery route (local or systemic)
**Target cell availability**	May not be many remaining supporting/hair cells/neurons to integrate with in profound SNHL	Therapy requires the presence of target cells; many genetic conditions destroy them early	Requires the presence of target cells and their responsiveness to drugs
**Precision of targeting**	Difficulty in ensuring the transplanted cells differentiate into correct phenotypes	Difficult to ensure the vector avoids off-target tissues	Diagnostics are needed to identify the underlying pathology of SNHL, to identify which drugs may work
**Engraftment and survival**	Variable integration of transplanted cells into cochlear structures remains a challenge	Currently relies on existing cell survival/cytoarchitecture	Concentration, dosing and side effects need to be assessed for each delivery route
**Functional integration**	Difficult to control whether new cells form functional synapses	Even if the gene is corrected, existing structural damage may prevent functional recovery	Functional restoration must be measurable
**Genetic complexity**	Applies regardless of genetic cause; not limited by mutation	Each therapy treats only a specific mutation or a small cluster of mutations	Underlying genetic factors likely impact the outcome, but are not well understood in the human population
**Vector/Carrier limitations**	Stem cells can elicit immune reactions; risk of clumping, misplacement, and uneven distribution	AAV vectors have small cargo limits; immune response to subsequent injections	Uptake and distribution concerns for intratympanic injections in different formulations
**Therapeutic window**	Low sensorineural plasticity over time	Must be delivered before permanent damage (often very early in life)	Identification and stratification of patients is essential
**Safety risks**	Tumour formation risk if stem cells are not fully differentiated; immune suppression or inflammation risk	Off-target genome editing; unintended immune responses; insertional mutagenesis	To be determined during clinical trials for each drug, combined with the chosen delivery mode
**Effect durability**	Unknown transplant survival time; potential degeneration	Unknown. Gene expression may wane over the years; unclear need for repeat dosing	Unknown. Needs to be optimised for systemic or local delivery strategy
**Species translation challenges**	Regeneration shown in animals does not necessarily translate to humans	The human cochlea is less accessible and larger than the mouse cochlea	The effect on animals does not necessarily translate to humans
**Manufacturing and cost**	Complex to scale cell production; expensive personalised products; quality assurance costs	Viral vector manufacturing bottlenecks: high per-patient cost, different vectors for each gene deficiency	Probably not significant and likely to be similar to ocular therapeutics; treatment cost may be high if a surgical procedure is required
**Ethical issues**	Use of embryonic stem cells; potential in utero interventions	Germline editing concerns if embryo or prenatal editing is attempted	Equitable access to drugs for all

## Data Availability

The original contributions presented in this study are included in the article. Further inquiries can be directed to the corresponding author.
